# Cushion Mechanism of Goat Hoof Bulb Tissues

**DOI:** 10.1155/2019/3021576

**Published:** 2019-11-06

**Authors:** Weijun Tian, Hai Liu, Qi Zhang, Bo Su, Wei Xu, Qian Cong

**Affiliations:** ^1^Key Laboratory of Bionic Engineering (Ministry of Education, China), Jilin University, Changchun 130022, China; ^2^China North-Vehicle Research, Fengtai District, Beijing 100072, China

## Abstract

The hoof bulb sections of white goats were observed via scanning electron microscopy and stereomicroscopy in order to explore the cushion mechanism in the bulb tissue microstructures of hoofed animals. The hoof bulbs consisted of multilayer tissues, including an epidermal layer, a dermal layer, and subcutaneous tissues from outside to inside. A bionic model based on hoof bulb tissue composite structures was built with a normal model as the control. The microcosmic mechanics of the bulb tissues was analyzed via the finite element method. Simulations showed that when the bionic model was impacted by the top plates at the speed of 1-10 m/s, stress was concentrated in the epidermal layer and uniformly distributed in the dermal layer and dermal papillae, which effectively reduced the impact onto the ground. The cornified epidermal layer can resist the instant impact onto the ground, while the dermal papillae embedded in the dermal layer can store, release, and dissipate the impulsive energy, and the three parts synergically act in the cushion.

## 1. Introduction

The feet of animals consist of numerous bones, muscles, ligaments, and joints and are the major part of weight bearing and motion. Feet mainly function as cushion and support and show unique functions and characteristics during the activities of animals owing to the special tissue structures and biomechanical properties [1].

During the moving process, feet are the only part where animals make contact with the ground, and due to the sudden variation of forces upon the contact, the instant force is several times larger than the weight [2, 3]. The percussive waves induced by the ground reaction are transferred from the feet through the limbs and to the trunk and then through the backbones to the brain. In the natural surroundings, these animals usually go through a lot of uneven ground, which produces the large impact to the animal's body. Excessively large impact will severely damage the body and brain of animals [4]. The reason these animals adapt to the uneven ground is largely the protection of their foot structure.

The foot pads and claws are a great design of nature and are the protective and bearing structures of feet. The feet of camels are like tires filled with lipids [5]. The foot pad of camels consist of several layers, including the sole (cornified pad), common coverings, digital cushions, and yellow bed [6]. The broad and fat pads connecting the toes prevent camels from sinking into the loose sand and allow them to walk or run nonstop in deserts [2]. Their unique limbs make African elephants the terrestrial organism that can support the heaviest weight. In addition to other morphological characteristics, the feet of African elephants are equipped with large pads, which can bear, store, or absorb machinery force and are critical in force allocation [7]. The foot pads of African elephants are mainly composed of sheet-like or bunch-like fiber connective tissues, forming large gaps among metacarpals, metatarsal bones, and phalanges, and the gaps are filled with lipid tissues, and the inner of foot pads consist of collagens and web-like elastic fibers [8]. Histologically, the claws and foot pads are separated by the soft honeycomb-like bodies built by closed partitions or the collagen-strengthened elastic dissepiments and are filled with lipid (lipid tissue) cells [2, 9, 10]. Foot pads are excellent weight-bearing biostructures and depend on the closed cells filled with liquids or lipid tissues to cushion the large distortion. During deformation, the amount of such structural response is nonlinear [11]. During the past decades, many biomechanical studies have been conducted on animal claws and foot pads but are focused on the mechanical properties. In particular, for hoofed animals, much research is focused on the material structures, mechanical performances, and friction performance of horse hoofs [12–14].

The relationship between horse hoof wall composite structures and functions were investigated via morphological and mechanical studies [15, 16]. Morphological studies showed that the sizes, shape, and helical intermediate filaments of horse hoof walls can guide the growing direction of hoof wall cracks, so as to inhibit the inward and upward crack expansion mechanisms [17–19].

White goats, also called domestic goats, are the most numerous and widely distributed. With strong adaptability and surviving ability, they can survive under harsh cliff conditions. The living environment makes white goats extremely agile and strong. During foraging, they have to leap among cliffs. To avoid chase by natural enemies, they have to be fast and continuously jump down from dozens of meters high. Goat and blue sheep are cloven-hoofed animals, and the hoofs play important roles in its locomotion [20]. Goats have a rough soft and flexible pad on the bottom of their two-toed hoofs, and it is a shock absorber that also generates a friction force due to its texture [21]. The rear end of the hoof is wide and roughly spherical, and the outside of the toe is higher than the inside, so that the soil can be consolidated below the hoofs [22]. All these actions cannot be made by other animals of similar body form. In this study targeted at white goats, the unprocessed hoof bulb tissues were observed under scanning electron microscopy (SEM). Then, the bulb tissues were stained and histologically tested under microscopy. Finally, the biomechanical functions of the bulb tissue microstructures were simulated with the finite element method (FEM), and the cushion mechanism of hoof bulb tissues was elaborated.

## 2. Materials and Methods

### 2.1. Ethical Declaration

This study was approved by the Review Board of Jilin University (Changchun, China).

**Figure 1:**
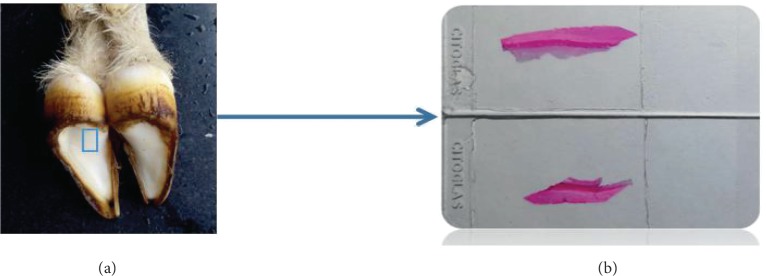
Hoof bulb tissues. (a) The location of the slices on the goat hoof and (b) the prepared goat hoof slices.

In total, 6 adult and healthy white goats without limitation to gender (20.0 ± 0.5 kg) were bought from a farmer in the suburb area of Changchun. The goats were anesthetized and killed via carotid bleeding. From 4 of the goats, the hoof capsules and flesh hoofs were collected. Then, bulb tissues of 1 cm^3^ were cut off (Figure 1) and fixed in 4% paraformaldehyde for 1 week. After that, the bulb tissues were put into a softening solution (75 mL of 60% ethanol and 25 mL of glycerol) for 1 month. The softened bulb tissues were dehydrated in an ethanol gradient, then treated in a dimethylbenzene solution until transparency, immersed in wax, and embedded in an MNT cool-hot embedder (SLEE, Germany) into paraffin bulks. The bulks were cut by a CUT5062 segment device (SLEE) into continuous tissue slices (5 *μ*m), which were then spread in a water bath at 40°C, sticked, and roasted in a roasting machine. After staining with hematoxylin-eosin (HE), Weigert-VG, and Sacpic, the slices were observed under a right-above fluorescence microscope (Axio Imager A2, Zeiss).

From the remaining 2 goats, the hoofs were cut off, and the hoof capsules, which can be easily contaminated on the surface, were pretreated. The hoof capsules were first washed with deionized water, then dust-removed, naturally dried, and washed with acetone and anhydrous ethanol to remove the surface oil stains and other contaminants. After that, the hoofs were dissected to acquire the hoof capsules, which were cleaned and frozen for use. The complete hoof capsules were taken out of the refrigerator, and after thawing and softening, were washed with clean water and wiped with dry towels. Then, the hoof capsules were cut off with scalpels. Sections about 5 mm wide, 5 mm high, and in the bulb wall thickness were extracted and placed onto glass slides, which were put in a cool place for natural drying. After that, the sections were stuck via electric plating adhesion onto a sample table, which was placed in vacuum film deposition equipment for gold spraying for 30 s, followed by observation under an EVO 18 scanning electron microscope (SEM, Carl Zeiss Microscopy GmbH, Jena, Germany).

### 2.2. Microscopic Observation

#### 2.2.1. SEM

**Figure 2:**
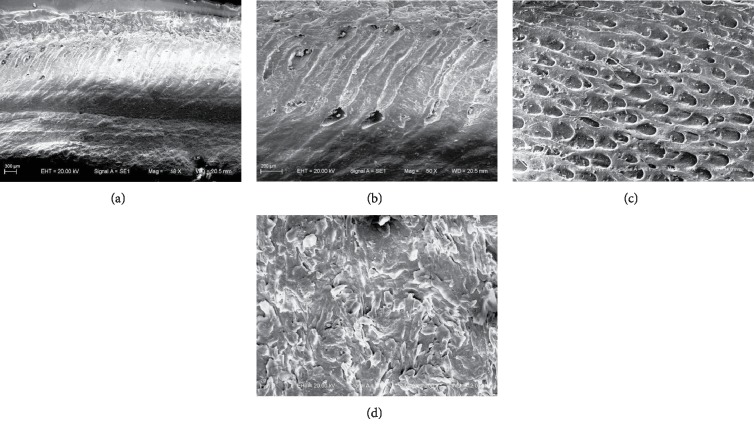
SEM images of hoof bulb tissues. (a) Longitudinal sections of bulb tissues; (b) longitudinal sections of the dermal layer; (c) cross-sections of the dermal layer; (d) cross-section of the epidermal layer.

**Figure 3:**
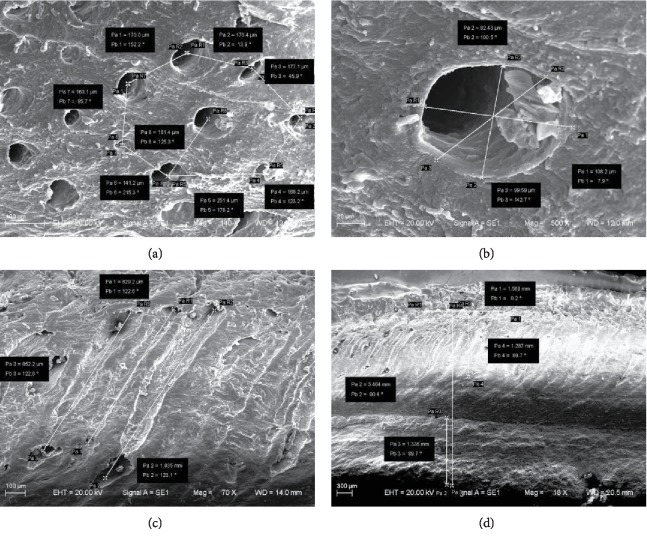
The measurement of the tubes by SEM: (a) the tube spacing (b) the diameter (c) the inclined angle, and (d) the thickness.

The hoof bulb tissues were observed with SEM. The microstructures (Figure 2(a)) show that the longitudinal sections of hoof bulb tissues can be divided into two layers. The upper layer consists of many honeycomb-like inclined fine tube structures. As shown in Figure 3, the parameters of the tubed were measured and evaluated, which would be used in the simulation. The tubes are separated at a distance of 140-250 *μ*m (Figure 3(a)), the diameters are 80-110 *μ*m (Figure 3(b)), the inclined angle is about 57° (Figure 3(c)), and the layer thickness is about 1.3 mm (Figure 3(d)).

The epidermal layer is fully cornified (the very hard and cornified layer in contact with the ground (together with nails)), and the relatively smooth and even cornified epidermis grows in a cascaded way and contains cracks. The layers are arranged in parallel and the plys are connected tightly (the cornified epidermis layer in Figure 2(d) is superimposed from tissue structure sheets). No defects or holes were found (Figure 2(d)).

#### 2.2.2. Histologic Examination

**Figure 4:**
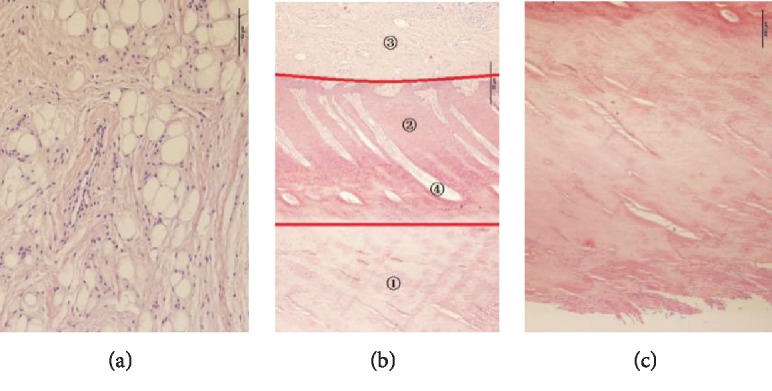
Histological images of hoof bulbs: (a) subcutaneous connective tissues, (b) longitudinal sections of bulb tissues, and (c) epidermis layer. ①: epidermal layer; ②: dermal layer; ③: subcutaneous connective tissues; ④: dermal papillae.

The stereo microscope showed that the hoof bulb tissues of white goats were divided from down to top into the epidermal layer (①), dermal layer (②), dermal papillae (④), and subcutaneous connective tissues (③) (Figure 4(b)). The epidermal layer was fully keratinized (Figure 4(c)), which thickened the hoof bottom. This structure helps to protect the hoof capsules and avoids microbial invasion and, when abrasion from the environment is intensified, can effectively minimize injuries to the hoofs [23]. Abundant fat lobules were found in the bulb tissues, and each lobule contained numerous fat cells and was surrounded mainly by loose connective tissues and contained abundant collagenous fibers, elastic fibers, and arteriovenous tallies (Figure 4(a)).

### 2.3. Finite Element Method (FEM) Modeling and Simulation

**Figure 5:**
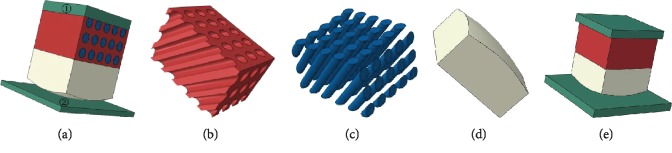
(a) Hoof bulb tissue assembly model, (b) dermal layer model, (c) dermal papilla model, (d) epidermal layer model, and (e) normal model. ①: top plate; ②: ground supporting plate.

An FEM model based on the hoof bulb tissue microstructure was built and used to study the biomechanical functions of this composite structure. A composite microstructured bionic model was established with the 1/250 hoof bulb tissues as the prototype. This bionic model consisted of three parts: the cube with inner inclined fine tubes, the inclined cylinder, and the cube with one arc surface, which represented the dermal layer (Figure 5(b)), dermal papillae (Figure 5(c)), and epidermal layer (Figure 5(d)), respectively. For the dermal papilla model, the tube space was set as 160 *μ*m, diameter was 80 mm, incline angle was 50°, and layer thickness was 1.3 mm. A rectangular plate was connected to the top of the bionic model and was called the top plate (①) (Figure 5(a)), which represents the goat body with mass and velocity. In the simulating process, the top plate acts as the dynamic body inducing crash to the model, simulating the actual crash coming from the goat body to goat hoofs. Another rectangular plate was fixed at the lower part of the bionic model to simulate the hard ground (②) (Figure 5(a)). A 3D model involving all the layers of the bionic model was built and ground on the FEM software ABAQUS. Through dynamic simulation and analysis on ABAQUS, the walking or running of white goats was simulated as well as the process of hoof capsule bulbs contacting the ground. The organism soft tissues are usually manifested as nonuniform, anisotropic, pseudoincompressible, and nonlinear-plastic-viscoelastic materials [24]. To simplify the complex models, researchers have idealized the foot bottom tissues as homogeneous and isotropic linear elastic materials [25]. The material properties of different parts of the hoof bulb tissues as well as the elements of FEM division [26–28] are shown in Table 1. The material properties of the normal model are shown in Table 2. The mesh size of FEM was set as 0.02 mm.

When the mass or sizes of the object differed, the object with a smaller mass to contact surface area ratio is less affected by impulsive stress, because when the object crashes unto the ground under the same velocity conditions, the conclusion above will be obtained by the following formula:

(1) $$\begin{array}{c} \Delta P=F\cdot t=M\cdot \left(V_{1}-V_{2}\right),\;V_{2}=0, \end{array}$$

(2) $$\begin{array}{c} F=M\cdot \frac{V_{1}-V_{2}}{t},\;P=\frac{F}{S}=\frac{M}{S}\cdot \frac{V_{1}-V_{2}}{t}. \end{array}$$

**Figure 6:**
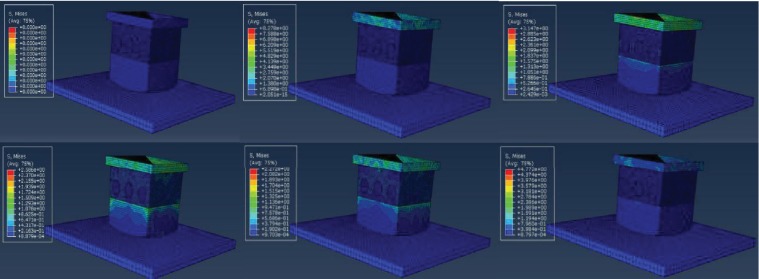
The simulation process of the bionic model.

In the formula, *V*_1_ represents the initial velocity exerted on the top plate and *t* is the time from the beginning of crash to the top plate velocity becoming zero. *M* is the mass of the top plate, *P* represents the impulse of the model under the crash of the top plate. With the same initial velocity, usually, we consider (*V*_1_ − *V*_2_)/*t* as the constant between the bionic model and the normal model. The smaller the mass to contact surface area ratio (*M*/*S*) is, the smaller the impulsive stress *P* is. The bionic model for simulation was a small part of the real hoof bulb tissues, the mass of the top plate is much smaller compared to the actual goat body mass. According to formula (2), when *M* is smaller, the stress is smaller so that the simulating comparing result is not obvious enough. To improve the simulation veracity to the actual situation, we set the initial velocity of the top plate in the FEM simulation as 1-10 m/s, which is larger than the velocity when the goat foot touched the ground; the actual crashing velocity of a goat is about 0.69 m/s-0.9 m/s according to our experiment high speed photography videos. This makes the simulating result more obvious. As shown in Figure 6, stress first occurs at the top plate, which indicates that the top plate is the moving plate.

To study the cushion mechanism of goat hoof bulbs, a normal model was needed in addition to the bionic model. In the normal model, the dermal papillae and the dermal layer were considered as one body, and thus, this model only consisted of two layers, including the dermal layer and the epidermal layer (Figure 5(e)). For both the bionic model and the normal model, the elastic modulus, Poisson's ratio, and density all differed among different layers, but these models were composed of homogeneous, isotropic, and linear elastic materials. In the two models, the same initial velocity and restraint were loaded and then the outputs were compared.

## 3. FEM Result and Analysis

The process of the goat hoof bulbs contacting the ground was simulated on ABAQUS. As shown in Figure 6, in the simulating process, the instantaneous initial velocity is exerted on the top plate firstly. Then, the top plate transfers the energy to the simulating model. So the stress first occurs on the top plate. With the simulating process ongoing, the other parts of the simulating model will have stress and stain corresponding to the impact from the top plate,. so that the top plate's stress will change constantly during the whole simulation (Figure 6). The bionic model and the normal model were separately fixed onto the supporting plates on the ground, and the top plates were assigned with the initial impact velocity of 1-10 m/s, and the models outputted the impact force onto the supporting plates (ground reaction), displacement of the upper surface of the dermal layer, and the inner stress of the models (Figure 6).

### 3.1. Ground Reaction

**Figure 7:**
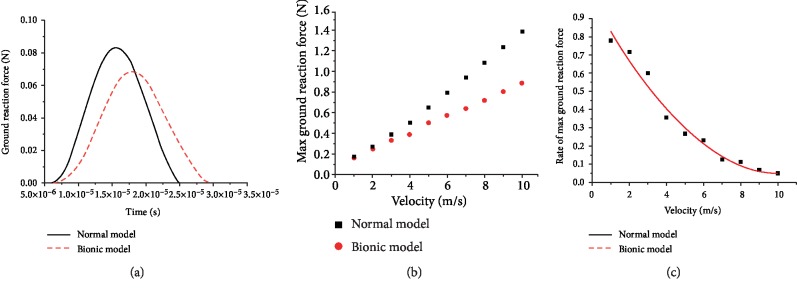
(a) Ground reactions from the bionic model and the normal model when the top plates were loaded with the velocity of 1 m/s; (b) peak ground reactions from the two models under the loaded velocity of 1 to 10 m/s; (c) ratio of reaction peak of the bionic model to the normal model under the loaded velocity of 1 to 10 m/s.

During the motion of a goat, the reaction when the hoof bulbs contact the ground is one of the important indices to measure the biomechanical performance of hoof bulb tissues. As shown on the reaction-time curves (Figure 7(a)), the reaction from the supporting plates of the two models changed with time in a typical single peak mode, indicating the ground impact during the contact between the hoof bulbs, and the ground impact gradually intensified and then weakened. The ground contact time of the bionic model was longer than that of the normal model (3 × 10^−5^ vs. 2.5 × 10^−5^ s), and the ratio of the peak reaction of the bionic model to the normal model was 0.85 (0.072 vs. 0.084 N). As the loading velocity on the top plates rose, the peak reaction from the two models both linearly increased and the increasing rate was larger in the normal model (Figure 7(b)). As the loading velocity on the top plates rose from 1 to 10 m/s, the ratio of peak reaction from the bionic model to the normal model declined from 0.85 to 0.62, and the changing rate decreased (Figure 7(c)).

### 3.2. Displacement of the Upper Surface

The goat hoof bulb hypodermic tissues are rich in fat tissues and elastic tissues, which largely reduce the pressures upon contact with the ground and can scatter various external forces imposed on the hoofs during the walking process. The deformation of hoof bulbs is another key indicator of biomechanical performances of hoof bulb tissues. During the FEM analysis, since the volumetric change rate of models can be hardly computed, the displacement of the upper surface was used to characterize the deformed amount of hoof bulb tissues.

**Figure 8:**
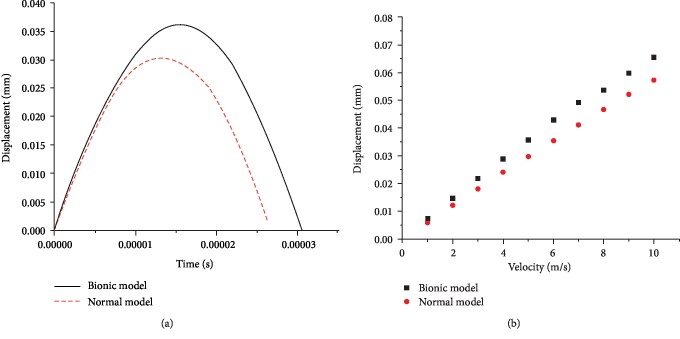
(a) Displacement on the upper surface of the bionic model and the normal model at the loading velocity of 5 m/s; (b) peak displacement on the two models at different loading velocities.

When the loading rate on the top plates was 5 m/s, the maximum displacement on the upper surface of the bionic model and the normal model was 0.36 and 0.30 mm, respectively, and the displacement-time curves were both typical of the single peak mode. At the early stage upon the impact onto the supporting plates (5 × 10^−5^ s), the displacements of the two models were nearly the same, but with the prolonging of time, the difference of displacement between the two models gradually enlarged and finally stabilized at 0.09 mm (Figure 8(a)). As the loading velocity on the top plates increased, the maximum displacements of both models increased and the difference between the two models was unchanged.

### 3.3. Internal Stress Distribution in the Epidermal Layer Model

**Figure 9:**
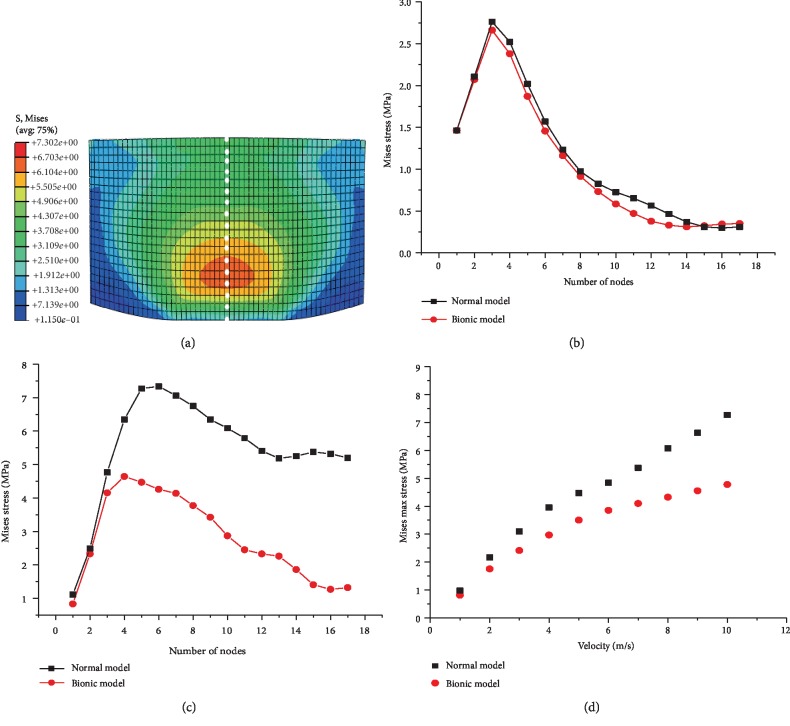
(a) Stress nephogram on the longitudinal section of the epidermal layer; stresses at different nodes when the loading speed onto the top plates was (b) 1 m/s or (c) 10 m/s; (d) peak internal stress on the epidermal layer in both models at different loading speeds.

Since only the epidermal layers were completely the same in the two models, when the ground reaction during the contact with the ground maximized, the stress magnitudes and distributions on the central axis of the epidermal layers both differed between the two models. Along the central axis of the epidermal layer, 17 grid nodes were marked from down to top to extract the stresses (Figure 9(a)).

Clearly, when the ground reaction maximized, the stress was concentrated on the epidermal layer, which mainly occurred in the lower part of the epidermal layer (Figure 9(a)). During the impact onto the ground, when the ground reaction maximized, the stress on the epidermal layer from the down to the top first increased and then decreased in both models (Figures 9(a)–9(c)). When the loading velocity was 1 m/s, the stress-node curves of the two models nearly overlapped, as the stress both increased from 1.5 to 2.755 and then slowly declined to 0.25 MPa, indicating that at low-velocity impact, the stress magnitude and distribution on the epidermal layer were identical between the two models (Figure 9(b)). When the loading velocity was 10 m/s, the stress of the normal model rose from 2.5 to 7.5 and then declined to 5.5 MPa, while that in the bionic model first increased from 2.25 to 5.25 and then slowly declined to 2.5 MPa, indicating the stress distributions on the epidermal layers were identical between the two models (Figure 9(c)). As the loading velocity rose, the stresses at the lower parts were identical between the two models, but at the upper nodes, the stress differences between models were enlarged (Figures 9(b) and 9(c)). As the loading velocity on the top plates rose from 1 to 10 m/s, the ratio of peak epidermal stress from the bionic model to the normal model increased from 1.0 to 1.45 (Figure 9(d)).

### 3.4. Stress Distributions inside the Bionic Model

The goat hoof bulb tissues consist of dermal papillae, the dermal layer, and the epidermal layer. The epidermal layer is fully cornified and mainly functions to thicken the hoof bottom. When the environmental abrasion is intensified, the epidermal layer can effectively minimize the injuries to the hoofs. Dermal papillae enlarge the connection area between the epidermis and the derma, which contributes to the firm connection between the two.

**Figure 10:**
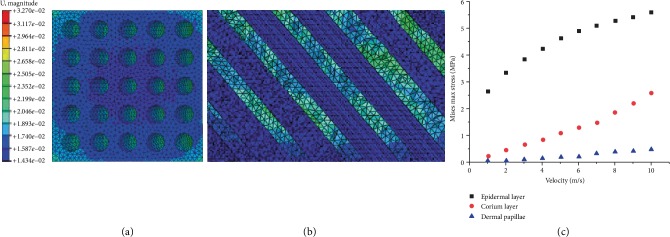
(a) Cross-sectional stress nephogram at the dermal layer of the bionic model; (b) longitudinal-section stress nephogram at the dermal layer of the bionic model; (c) peak internal stresses of dermal papillae, dermal layer, and epidermal layer at different loading velocities.

With FEM, the process of hoof bulbs impacting the ground was simulated. The peak stress and stress nephograms of dermal papillae, the dermal layer, and the epidermal layer at different impacting velocities were detected (Figure 10).

When the ground reaction peaked, the internal stress distributions in the dermal papillae and the dermal layer of the bionic model were both uniform (Figures 10(a) and 10(b)). When the loading velocity on the top plates rose from 1 to 10 m/s, the peak stress increased from 0.038 to 0.474 MPa in the dermal papillae, from 0.29 to 2.58 MPa at the dermal layer, and from 2.66 to 5.6 MPa at the epidermal layer. As the loading velocity increased, the changing rate of the internal peak stress in the dermal papillae of the bionic model was unchanged, that in the dermal layer gradually increased, but that in the epidermal layer gradually declined.

## 4. Discussion

SEM and histological observation showed the goat hoof bulb tissues mainly consist of an epidermal layer, a dermal layer, and hypodermic connective tissues. The epidermal layer is fully cornified and is the hardest part of hoof bulb tissues and mainly functions to thicken the hoof bottom and directly contacts with the ground. During motion, when the impact between the hoofs and the ground was aggravated and the environmental wear was intensified, the injuries to the hoofs can be effectively decreased. The dermal layer is rich in fat tissues and elastic fibers, which largely reduce the pressures upon contact with the ground and can scatter the various external forces imposed onto the hoofs during the walking process and thus are critical in preventing damage to the hoofs. Dermal papillae enlarge the connection area between the epidermis and the derma, which contributes to the firm connection between the two. The dermal papillae are regularly buried in the dermal layer at the inclining angle of 57°, and the dermal papillae of hoof bulb tissues are much longer and more regularly distributed than those in the skin.

FEM showed the bionic model based on the dermal layer microstructures can well reduce the ground reaction, and this ability within a certain range was gradually strengthened with the increment of loading velocity. This was because the epidermal layer, the dermal layer, and dermal papillae were different in structures and material properties and can synergistically act to weaken the ground impact. During the collision process, the epidermal layer experienced small deformation and internal stress concentration and thus undertook a large portion of the impact. The dermal layer is very soft and can absorb the impulsive force by generating very large deformation. The dermal papillae regularly arranged at the inclination angle of 50° are very soft and can effectively reduce the transfer of impact from the epidermal layer to the dermal layer and also disperse the impact in the dermal layer, which avoids the stress concentration and thereby prevents the dermal layer from being destroyed. Based on the foot cushion property research on other animals like the cat's paw, the impact to the ground is usually reduced by the material characteristics of fat [29, 30], whereas the cushion mechanism of the goat hoof is determined by the composite structure and inclined holes, not only the materials. This composite structure with holes can produce more obvious cushion capacity by the coupling methodology (multilayer structure and hole structure). According to some researches about the horse hoofs, the tubules also exist at the inner hoof wall. The inclined angle of the tubules is different between the left and the right foot, arranging from 86.47° to 104.85°; the tubule spacing ranges from 0.36 to 0.53 mm; and the diameters are about 0.2 mm [31, 32]. The inclined angle of the goat hoof inner wall is about 57°, which is smaller than the horse. And the tubes of the goat hoof wall are separated at a distance of 0.14 mm-0.25 mm with 0.08-0.11 mm diameter; the separated distance and diameter are all smaller than those of the horse. We consider that the difference is related to the different living surroundings of goats and horses: the horse usually runs on the prairie and the crash condition to the hoof is the soil, whereas for the goat, the crash condition is usually the rock, which is harder than soil, so that the goat may need more cushion capacity on their hoofs, and the goat hoofs having better cushion capacity than horse hoofs result from the differences in the tubules' inclined angle, density, and diameter.

As the collision speed increased, the three layer structures of hoof bulb tissues showed different biomechanical characteristics. As the speed rose, the impact onto the bulb tissues was intensified accordingly, but the peak inner stresses of the epidermal layer, dermal layer, and dermal papillae increased at different rates. The increasing rate of internal peak stress declined at the epidermal layer, rose at the dermal layer, and was unchanged in the dermal papillae. It indicated that the special microstructure layer can automatically adjust the impact absorption ratios among different layers during the different ground impacts. During low-speed collision, the hoof bulbs are affected by low ground impact; the epidermal layer including the cuticle layer is thick and hard enough, so it can suffer from the stress and protect the inner part of the hoof from being damaged. So the epidermal layer undertakes the majority of the impact. As the collision speed increases, the collision and friction between the epidermal layer and the ground are aggravated, which will further damage the epidermal layer, so the epidermal layer suffers from a smaller portion of the impact (as shown in Figure 10(c)) to protect itself from being damaged. It indicates that when the impact between the hoofs and the ground was aggravated and the environmental wear was intensified, the injuries to the hoofs can be effectively decreased because of the lower damage of the epidermal layer. This special microstructure has excellent cushion ability to effectively reduce the ground impact onto the trunk and prevents animals from being damaged by high-strength movement, such as running and jumping.

### 4.1. Study Limitation

According to results of the FEM simulation, the study emphasizes the cushion mechanism of the goat hoof bulb tissue structure and the model is established with three structures with different material properties. But the material of each bionic structure is set as homogeneous and isotropic linear elastic materials. The pressure distribution may be different compared to the model with nonuniform, anisotropic, pseudoincompressible, nonlinear-plastic-viscoelastic materials. The model in this study just discusses the results of a homogeneous and isotropic linear elastic material model. Further, we can research about the nonuniform, anisotropic, pseudoincompressible, and nonlinear-plastic-viscoelastic material model. Based on comprehensive consideration, in this article, the FEM model size is set to 0.02 mm to investigate the cushion capacity of the model. However, the convergence may become different if the FEM model size is changed. In the future study, we will design different FEM mesh size models and analyze the different types of convergence. Considering the limitation of simulation, we will design the physical model according to the designed model and implement the material object experiment, to verify the simulating result by comparing to the material object experiment.

## 5. Conclusion

SEM and histological observations show that the goat hoof bulb tissues mainly consist of an epidermal layer, a dermal layer, and hypodermic tissues. The dermal layer contains many honeycomb-like inclined fine tubes, and the columnar dermal papillae are buried inside. The full-cornified epidermal layer is located at the lower part of the dermal layer and grows in a cascade-like way and is relatively smooth and even. The epidermal layer, dermal layer, and dermal papillae synergistically act to get excellent cushion ability. The epidermal layer, dermal layer, and dermal papillae of the composite structure can automatically adjust the impact absorption ratios among layers during the ground impact. According to the simulation results, the cushion capacity increases with the increase of the collision velocity. When the collision velocity increases, the internal peak stress changing rate is different among the components of the bionic model; the changing rate of the internal peak stress in the dermal papillae is unchanged, gradually increased in the dermal layer, but gradually declined in the epidermal layer. The study on biomechanical characteristics of goat hoof bulb tissues shows mechanical action in the special hoof microstructure of hoofed animals and offers new clues for the design of cushion pads.

## Figures and Tables

**Table 1:** Material properties and element types of the FEM model.

Name	Element	Young's modulus *E* (MPa)	Poisson's
Epidermal layer	3-D tetrahedrons	113.8	0.38
Dermal layer	3-D tetrahedrons	7.0	0.45
Dermal papillae	3-D tetrahedrons	0.6	0.495
Top plate	3-D tetrahedrons	206 × 10^3^	0.3
Ground supporting plate	3-D tetrahedrons	206 × 10^3^	0.3

**Table 2:** Material properties and types of the normal model.

Name	Element	Young's modulus *E* (MPa)	Poisson's
Epidermal layer	3-D tetrahedrons	113.8	0.38
Dermal layer	3-D tetrahedrons	7.0	0.45
Top plate	3-D tetrahedrons	206 × 10^3^	0.3
Ground supporting plate	3-D tetrahedrons	206 × 10^3^	0.3

## Data Availability

The data used to support the findings of this study are available from the corresponding author upon request.
